# Take a look at plant DNA replication: Recent insights and new questions

**DOI:** 10.1080/15592324.2017.1311437

**Published:** 2017-04-04

**Authors:** Savannah D. Savadel, Hank W. Bass

**Affiliations:** Department of Biological Science, Florida State University, Tallahassee, FL, USA

**Keywords:** Chromatin, DNA synthesis, maize, omero, rDNA, 3D microscopy

## Abstract

Recent advances in replicative DNA labeling technology have allowed new ways to study DNA replication in living plants. Temporal and spatial aspects of DNA replication programs are believed to derive from genomic structure and function. Bass et al. (2015) recently visualized DNA synthesis using 3D microscopy of nuclei at three sub-stages of S phase: early, middle and late. This addendum expands on that study by comparing plant and animal DNA replication patterns, by considering implications of the two-compartment model of euchromatin, and by exploring the meaning of the DNA labeling signals inside the nucleolus. Finally, we invite the public to explore and utilize 300 image data sets through OMERO, a teaching and research web resource for visualization, management, or analysis of microscopic data.

Temporal and spatial aspects of DNA replication programs are tightly coupled to genomic structure and function, as evidenced by decades of research and summarized by reviews^1-4^ on replication timing studies and observations primarily from animal cells. In plants, recent advances in DNA labeling technologies have created exciting new opportunities to study DNA replication in nuclei from naturally developing organs.^5^ Bass et al. (2015)^6^ used 3D microscopy to examine spatiotemporal patterns of DNA replication in maize and found that the patterns were quite different from those described for mammalian cells, specifically at middle S-phase. This addendum to that study^6^ offers additional insights, speculations, and questions relating to three topics; the difference in plant versus animal replication timing patterns, the predictions made by the mini-domain chromatin fiber replication timing model, and the basis for punctate DNA synthesis inside the nucleolus. In addition, we describe a new public 3D image database (OMERO) housing previously unavailable 3D data sets.

## Plants and animals show different replicative labeling patterns in middle S-phase nuclei

**Figure 1.**
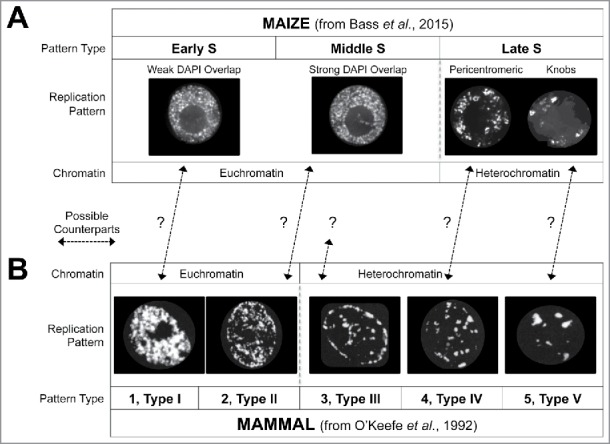
Cross-kingdom comparison of temporal patterns of DNA replication. (A) Maize nuclei from early, middle, or late S-phase, showing representative examples from maize EdU-labeled root-tip nuclei (from Bass et al.^6^). (B) Chinese Hamster Ovary nuclei from 5 sequential stages of S-phase, showing BrdU-labeled cell culture nuclei (from Fig. 4 of O'Keefe et al., 1992^10^). The replication pattern types are labeled as “1–5” (O'Keefe et al.^10^) and also “type I-V” (From Zink 2006^1^). The question marks reflect untested counterpart assignments.

Previous spatial and temporal patterns of DNA synthesis has been reported for plants.^7-9^ The maize root tip study allowed for a close comparison between the spatiotemporal replication patterns of maize vs. those of mammals, organisms with genomes of comparable size and complexity. A summary of the plant (maize, root tip nuclei) vs. animal (hamster, CHO cell culture nuclei) patterns is shown in Fig. 1. We noted a remarkable difference between the maize middle S-phase pattern and the canonical mammalian middle S-phase pattern, called “3” by O'Keefe et al. in 1992^10^ or “type III” by Zink in 2006.^1^ In mammals, one observes middle S-phase DNA synthesis to be primarily confined to perinuclear and perinucleolar regions. This primarily peripheral staining is not observed in maize, which like other plants, lack highly conserved homologs of genes for animal lamin and lamin-binding proteins, as described by Ciska et al.^11^ Consequently, plants may lack some of the organizational properties or DNA sequences typically resident at the animal nuclear periphery.

Another question is whether or not the distinction between these maize patterns are peculiar to this species, or common to plants, or also found in animals. It remains possible that animal type 1 and 2 could be distinguished as two compartments, but this has not yet been tested using quantitative 3D correlation analysis. In the plant study, late S-phase nuclei often appeared as one of two sub-types (pericentromeric and knobs). These are shown side by side in Fig. 1, reflecting their presumed temporal order, but coming from a single flow-sorted “Late-S” gate (see Fig. 1C from Bass et al., 2015^6^), they cannot be ordered in time. Even so, they do bear some resemblance to the last two patterns, 4 and 5, in animals.

## The mini-domain chromatin fiber replication timing model raises numerous questions

**Figure 2.**
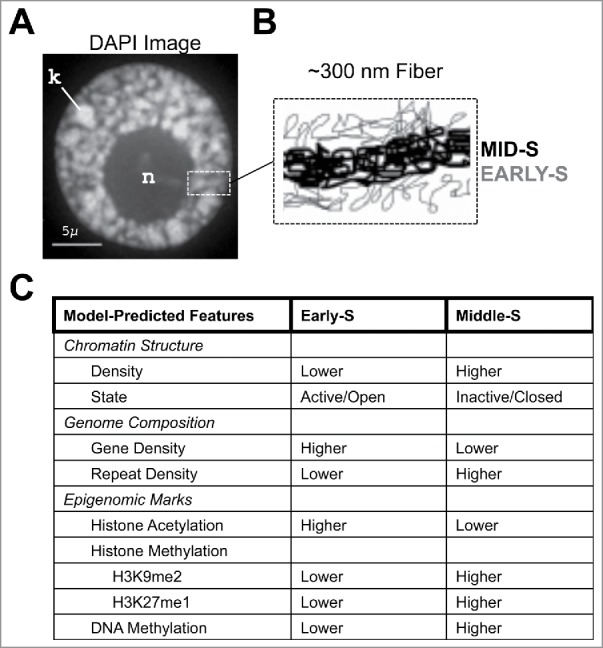
Predicted chromatin features based on the mini-domain chromatin fiber replication timing model. The maize interphase nucleoplasm was observed to be partitioned into 2 types of euchromatin, early-S or middle-S. (A) DAPI image of a maize nucleus at interphase showing a large nucleolus (n) and thick, ∼300 nm chromatin fibers (dashed box). (B) Diagram of the model showing the 2 types of euchromatin, MID-S (thick/black) and EARLY-S (thin/gray). (C) Table of chromatin features that might show differential assortment into the 2 types of chromatin on the basis of known or reported genomic features and epigenomic marks (as reviewed for example by Fuchs and Schubert^17^).

One of the unexpected discoveries from Bass et al.^6^ was the conclusion that maize euchromatin, and by extension that of other plants, may exist as intermingled mixtures of two compartments. This observation, congruent with the coexistence of multiple chromatin states, led to the model in which maize interphase nucleoplasm can be partitioned into two types of “euchromatin” - which we call early-S chromatin or middle-S chromatin, as diagrammed in Fig. 2. These two are experimentally distinguished by their condensation state and by their replication timing. One compartment is characterized as being preferentially replicated in early-S phase, lightly stained with DAPI, and comprised of low density chromatin presumably enriched in active genes. The other compartment, comprised of ∼300 nm fibers, is characterized as being preferentially replicated in middle S-phase, more heavily stained with DAPI, and comprised of higher density chromatin presumably enriched in repetitive or mobile DNA elements.

This two compartment mini-domain model raises questions and hypotheses (see Fig. 2C) for future research. Namely, is the early-S chromatin enriched in epigenomic marks typical of gene-rich areas with open or active chromatin? These marks should co-purify (ChIP assays) and co-localize (3D microscopy) with genomic sequences labeled at early S-phase. Likewise, is the middle-S chromatin enriched in epigenomic marks typical of repetitive sequences with closed or inactive chromatin? Moreover, these marks, such as H3K9me2 and DNA methylation,^12^ should co-purify and co-localize with genomic sequences labeled at middle S-phase, while also marking the even more condensed constitutive heterochromatin found in late S-phase chromatin. Another extension of this model is that plant species devoid of repeat intergenic DNA may lack a cytological pattern like that seen for maize middle-S. Consistent with this are the findings that describe replication as exhibiting two major phases on the basis of genomic^13^ or cytological^9^ analyses in Arabidopsis, a small genome plant notably devoid of repetitive DNA.

Further questions address the nature of early-S vs. middle-S chromatin. Are these two types of euchromatin stable across cell types and development, or do they have the capacity to be changed? And to what extent does transcriptional activity govern replication timing? For instance, do inactive genes replicate at middle S-phase? Conversely, do transcriptionally active repeat sequences replicate early? It is clear that in yeast and animals replication timing is generally coordinated with transcriptional competence,^14^ but these principles remain largely unexplored in plants. Finally, do boundaries or barriers that can temporarily stall replication forks exist so as to delimit early from middle S-phase? And are there specific origins that fire in middle S-phase, or do the forks simply eventually progress into the middle-S chromatin? As an experimental system, the cytology of replication timing in maize offers new avenues of research, complementing and extending contemporary epigenomic research strategies.

## Punctate DNA synthesis occurs inside the nucleolus

**Figure 3.**
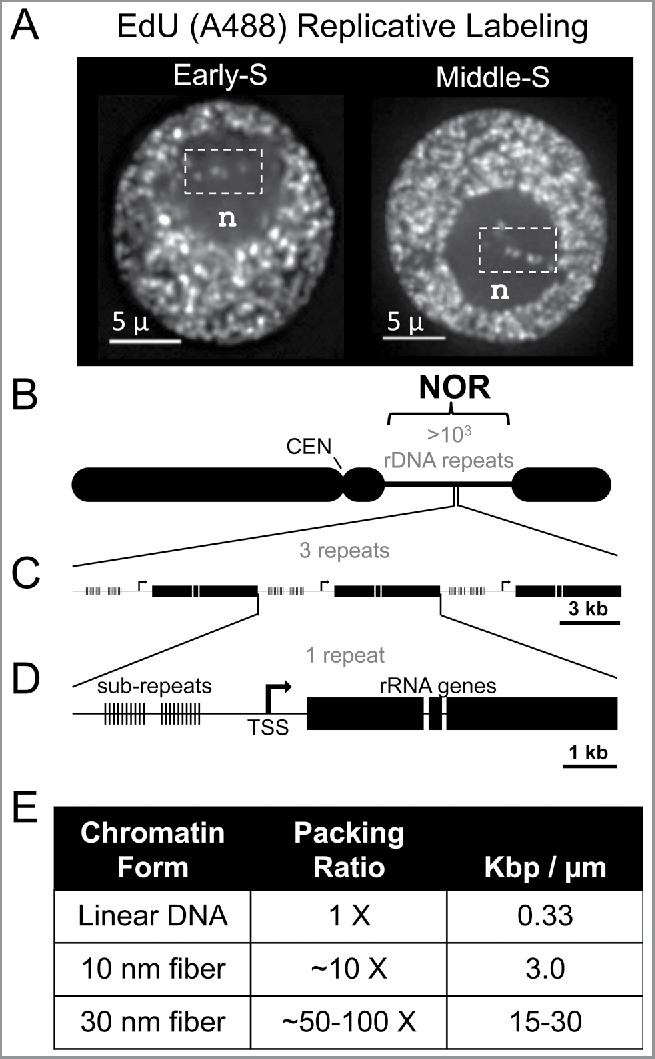
Replicative labeling signals can be seen inside the nucleoli as discrete punctate foci observed in early and middle, but not late S-phase nuclei. (A) Examples of EdU-labeled DNA replication from Early-S or Middle-S nuclei showing intra-nucleolar labeling (white dashed boxes). (B) Diagram of maize chromosome 6 showing the NOR with copy number estimate from Rivin et al.^18^ (C) Enlarged diagram of 3 rDNA repeats. (D) Enlarged diagram of one ∼10kb rDNA repeat showing the 200 bp sub-repeats (thin hatch lines, as per McMullen et al.^16^) and the major transcription start site (TSS). (E) Table relating real-space dimensions of DNA as a function of packaging, including Watson-Crick B-form DNA (Linear DNA), “beads-on-a-string” chromatin (10 nm fiber), and one of the basic higher order chromatin fibers (30 nm fiber).

Replicative labeling signals appear inside the nucleoli as discrete punctate foci in early-S and middle-S as shown in Fig. 3 (dashed boxes, panel A), but not late S (not shown, but see Bass et al., 2015^6^). The nucleolus affords exceptional cytological clarity for the visualization of in situ DNA replication. Detection of intra-nucleolar replicative labeling at both early and middle S raises the possibility that different regions within the ∼10 kb rDNA repeat, or different repeats within the array, may undergo programmed asynchronous replication while inside the nucleolus. Previous reports of replication fork barriers^15^ and sub-repeat chromatin^16^ are consistent with this speculation. The spacing of adjacent non-late replicative/EdU signals in the nucleolus could mark sites within or between the 10-kb repeats, depending on the packaging ratio of the chromatin fiber (Table in Fig. 3E).

## See the 3D data for yourself on OMERO, a 3D image database for public exploration

**Figure 4.**
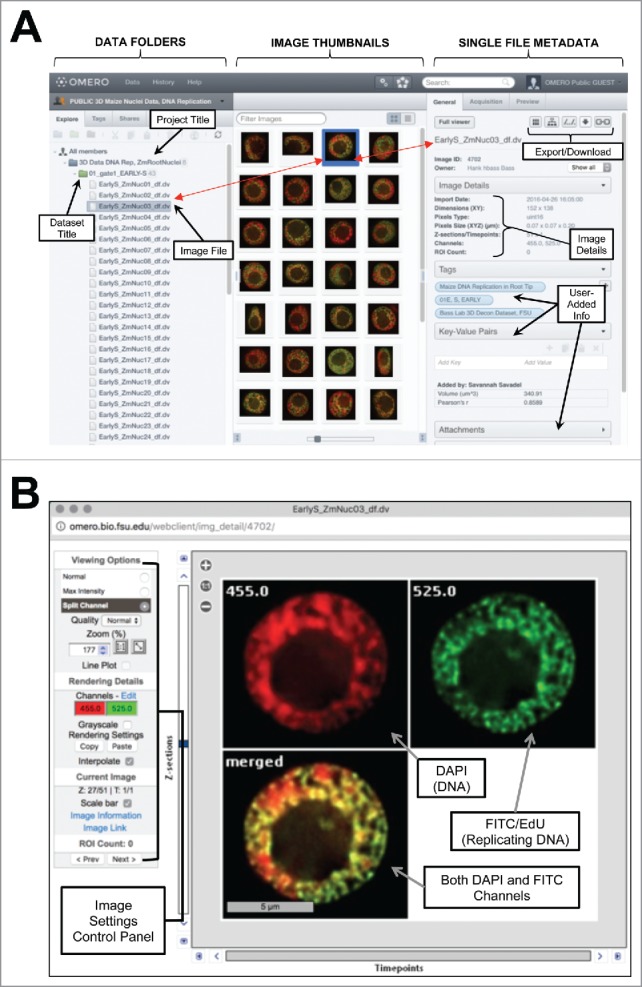
OMERO Image Database Example. (A) Screenshot of the file browser showing 3 partitions: DATA FOLDERS (Left) listing projects, data sets, and image files; IMAGE THUMBNAILS (Middle) displaying individual data sets; and SINGLE FILE METADATA (Right) displaying metadata and file specs auto-extracted during upload or user-added annotations such as tagging, value inputs, comments, or attachments. (B) Screenshot of the split channel view for a single nucleus. The image data display window and display settings control panel is shown. Basic controls allow for viewing as normal (single Z-sections), max intensity (through-focus projection), or split view (shown here). Display settings give user control over wavelength colors, brightness and contrast, scale bars, and image link for URL sharing.

To share DNA replication data more broadly for research and education, we have set up a public database at omero.bio.fsu.edu, housing hundreds of source images analyzed by Bass et al. in 2015.^6^ The OMERO platform uses open-source software and data format standards for the storage and manipulation of biologic microscopy data (www.openmicroscopy.org/site). Fig. 4 shows explanatory screenshots of the graphical web browser interface for organizing and viewing the image data. Detailed images with metadata can be exported to local files in their original or other (OME-TIFF, JPEG, PNG, or TIFF) formats for use with customized software. Here we invite scientists, teachers, and students to use this resource to explore published plant 3D replication data. Public access to the maize root tip replication image folders, in “3D Data DNA Rep, ZmRootNuclei,” https://goo.gl/CTI06F is available using the login “Public” and the password “omero,” with opportunities (contact HWB) for expanding to include other published plant cytogenetics images. As a working archive, the omero system solves a chronic limitation in the practice of sharing primary scientific and cytogenetic image data. The broader scientific community can explore, analyze, or even initiate new investigations using any or all of the 300 3D images of plant DNA replication. In conclusion, we have highlighted three of our favorite aspects of the recent maize root tip DNA replication study, emphasizing new questions, model predictions, and future research directions. These ideas, along with the public image database, provide new opportunities to leverage plant DNA replication studies to test and define structure-function relationships that underlie plant genome behavior.

## References

[1] Zink D The temporal program of DNA replication: new insights into old questions. Chromosoma 2006; 115:273-87; PMID:16552593; http://dx.doi.org/10.1007/s00412-006-0062-8 16552593

[2] Gilbert DM, Takebayashi SI, Ryba T, Lu J, Pope BD, Wilson KA, Hiratani I Space and time in the nucleus: developmental control of replication timing and chromosome architecture. Cold Spring Harb Symp Quant Biol 2010; 75:143-53; PMID:21139067; http://dx.doi.org/10.1101/sqb.2010.75.011 21139067

[3] Jackson D, Wang X, Rudner DZ Spatio-temporal organization of replication in bacteria and eukaryotes (nucleoids and nuclei). Cold Spring Harb Perspect Biol 2012; 4:a010389; PMID:22855726 22855726 10.1101/cshperspect.a010389 PMC3405862

[4] Dileep V, Rivera-Mulia JC, Sima J, Gilbert DM Large-scale chromatin structure-function relationships during the cell cycle and development: insights from replication timing. Cold Spring Harb Symp Quant Biol 2015; 80:53-63; PMID:26590169; http://dx.doi.org/10.1101/sqb.2015.80.027284 26590169

[5] Bass HW, Wear EE, Lee TJ, Hoffman GG, Gumber HK, Allen GC, Thompson WF, Hanley-Bowdoin L A maize root tip system to study DNA replication programmes in somatic and endocycling nuclei during plant development. J Exp Bot 2014; 65:2747-56; PMID:24449386; http://dx.doi.org/10.1093/jxb/ert470 24449386

[6] Bass HW, Hoffman GG, Lee TJ, Wear EE, Joseph SR, Allen GC, Hanley-Bowdoin L, Thompson WF Defining multiple, distinct, and shared spatiotemporal patterns of DNA replication and endoreduplication from 3D image analysis of developing maize (*Zea mays* L.) root tip nuclei. Plant Mol Biol 2015; 89:339-51; PMID:26394866; http://dx.doi.org/10.1007/s11103-015-0364-4 26394866 PMC4631726

[7] Sparvoli E, Levi M, Rossi E Replicon clusters may form structurally stable complexes of chromatin and chromosomes. J Cell Sci 1994; 107(Pt 11):3097-103; PMID:7699008 7699008 10.1242/jcs.107.11.3097

[8] Samaniego R, de la Torre C, Moreno Diaz de la Espina S Dynamics of replication foci and nuclear matrix during S phase in *Allium cepa* L. cells. Planta 2002; 215:195-204; PMID:12029468; http://dx.doi.org/10.1007/s00425-002-0741-5 12029468

[9] Yokoyama R, Hirakawa T, Hayashi S, Sakamoto T, Matsunaga S Dynamics of plant DNA replication based on PCNA visualization. Sci Rep 2016; 6:29657; PMID:27417498; http://dx.doi.org/10.1038/srep29657 27417498 PMC4945867

[10] O'Keefe RT, Henderson SC, Spector DL Dynamic organization of DNA replication in mammalian cell nuclei: spatially and temporally defined replication of chromosome-specific alpha-satellite DNA sequences. J Cell Biol 1992; 116:1095-110; PMID:1740468; http://dx.doi.org/10.1083/jcb.116.5.1095 1740468 PMC2289349

[11] Ciska M, Masuda K, Moreno Diaz de la Espina S Lamin-like analogues in plants: the characterization of NMCP1 in *Allium cepa*. J Exp Bot 2013; 64:1553-64; PMID:23378381; http://dx.doi.org/10.1093/jxb/ert020 23378381 PMC3617829

[12] West PT, Li Q, Ji L, Eichten SR, Song J, Vaughn MW, Schmitz RJ, Springer NM Genomic distribution of H3K9me2 and DNA methylation in a maize genome. PLoS One 2014; 9:e105267; PMID:25122127; http://dx.doi.org/10.1371/journal.pone.0105267 25122127 PMC4133378

[13] Lee TJ, Pascuzzi PE, Settlage SB, Shultz RW, Tanurdzic M, Rabinowicz PD, Menges M, Zheng P, Main D, Murray JAH, et al. *Arabidopsis thaliana* chromosome 4 replicates in two phases that correlate with chromatin state. Plos Genetics 2010; 6. 10.1371/journal.pgen.1000982 PMC2883604 20548960

[14] Rivera-Mulia JC, Buckley Q, Sasaki T, Zimmerman J, Didier RA, Nazor K, Loring JF, Lian Z, Weissman S, Robins AJ, et al. Dynamic changes in replication timing and gene expression during lineage specification of human pluripotent stem cells. Genome Res 2015; 25:1091-103; PMID:26055160; http://dx.doi.org/10.1101/gr.187989.114 26055160 PMC4509994

[15] Pasero P, Bensimon A, Schwob E Single-molecule analysis reveals clustering and epigenetic regulation of replication origins at the yeast rDNA locus. Genes Dev 2002; 16:2479-84; PMID:12368258; http://dx.doi.org/10.1101/gad.232902 12368258 PMC187456

[16] McMullen MD, Hunter B, Phillips RL, Rubenstein I The structure of the maize ribosomal DNA spacer region. Nucl Acids Res 1986; 14:4953-68; PMID:3725589; http://dx.doi.org/10.1093/nar/14.12.4953 3725589 PMC311503

[17] Fuchs J, Schubert I Chromosomal distribution and functional interpretation of epigenetic histone marks in plants In: Bass HW, Birchler JA, eds. Plant Cytogenetics. NY: Springer, 2012:231-53.

[18] Rivin CJ, Cullis CA, Walbot V Evaluating quantitative variation in the genome of *Zea mays*. Genetics 1986; 113:1009-19; PMID:3744025 3744025 10.1093/genetics/113.4.1009 PMC1202908

